# The Impact of the 10VIDA Program on Socioemotional Adjustment and Psychological Well-Being in Adolescents with Type 1 Diabetes Mellitus: A Preliminary Study

**DOI:** 10.3390/children12101291

**Published:** 2025-09-24

**Authors:** Pilar Rodríguez-Rubio, Javier Martín-Ávila, Esther Rodríguez-Jiménez, Selene Valero-Moreno, Inmaculada Montoya-Castilla, Marián Pérez-Marín

**Affiliations:** Department of Personality, Assessment and Psychological Treatments, Faculty of Psychology and Speech Therapy, University of Valencia, 46010 Valencia, Spain; mpiroru@alumni.uv.es (P.R.-R.); javier.martin@uv.es (J.M.-Á.); esther.rodriguez-jimenez@uv.es (E.R.-J.); selene.valero@uv.es (S.V.-M.); inmaculada.montoya@uv.es (I.M.-C.)

**Keywords:** 10VIDA, type 1 diabetes mellitus, adolescent, psychotherapy, chronic disease, emotional adjustment, psychological well-being

## Abstract

**Highlights:**

**What are the main findings?**

- **Effectiveness of the 10VIDA program**: Adolescents who participated in the intervention showed reductions in psychological discomfort, anxiety, depression, and perceived illness threat, as well as increases in self-esteem, emotional well-being, and socioemotional competencies. These effects were observed both in comparison with the control group and within the same adolescents over time. Improvements after 10VIDA intervention indicate that reshaping illness perception toward a more manageable and less threatening experience is possible through targeted psychological intervention.
- **Gender differences**: Girls reported higher levels of anxiety, depression, and illness perception, whereas boys demonstrated higher self-esteem, emotional well-being, and greater emotional regulation skills. These findings suggest that the psychosocial burden of T1DM is distributed unevenly between genders.
- **Parental influence and the importance of family support**: Adolescents who perceived greater parental support through autonomy promotion and open communication reported higher well-being, while perceptions of high psychological control correlated with emotional distress. This highlights the dual role of family dynamics in either buffering or intensifying the challenges of chronic illness.

**What are the implications of the main findings?**

- **Integration of psychosocial care into diabetes treatment**: The evidence supports the inclusion of structured psychological interventions, such as 10VIDA, in routine pediatric diabetes care. Such integration may enhance adherence to treatment regimens and ultimately improve both psychological and physical health outcomes.
- **Need for gender-sensitive interventions**: Because girls with T1DM appear more vulnerable to internalizing symptoms (e.g., anxiety, depression), interventions should include tailored components that specifically address these risks, while reinforcing boys’ emotion regulation skills to sustain positive adjustment.
- **Family-centered approaches**: The role of caregivers is critical. Programs that actively involve parents and other family members and try to promote characteristics such as autonomy and proper communication may strengthen resilience and reduce illness perceptions in adolescents.
- **Long-term and large-scale research**: Although preliminary results are promising, larger and more diverse samples are needed to confirm the effects. Future studies should assess whether these improvements in socioemotional adjustment translate into better glycemic control and long-term health outcomes. This can be done by measuring objective indicators like HbA1c across time to observe the degree to which it varies.
- **Contribution to intervention science**: By grounding itself in a theoretical model of clinical illness in adolescents that identifies the most important psychosocial factors in promoting a successful adaptation to illness, this intervention addresses previous gaps in psychoeducational programs. Its focus on emotional regulation, self-concept, and social relationships provides a theoretically consistent framework that could serve as a model for other chronic illness interventions.

**Abstract:**

**Background/Objectives**: Type 1 diabetes mellitus (T1DM) during adolescence increases the risk of psychosocial difficulties. To address these challenges, the 10VIDA program was developed to support psychological adjustment, treatment adherence, and quality of life. This study examined socioemotional factors linked to chronic illness adjustment and psychological well-being in adolescents with T1DM, assessing the impact of the 10VIDA intervention. **Methods**: Adolescents with T1DM participated in two studies: (1) an inter-group design with experimental and control groups assessed pre- and post-intervention, and (2) an intra-subject longitudinal design with three assessments (two pre-intervention and one post-intervention). All participants completed the 10VIDA intervention delivered online through seven sessions: five targeted adolescents with T1DM, while two included primary caregivers. The program’s main objective was to enhance well-being and quality of life. **Results**: Girls reported higher anxiety and depression, while boys showed greater self-esteem and emotional well-being. The experimental group experienced significant reductions in discomfort, anxiety, depression, and perceived illness threat, alongside improvements in self-esteem and psychological well-being. Intra-subject analysis also revealed increased well-being and decreased discomfort after intervention. **Conclusions**: The 10VIDA program effectively addressed socioemotional challenges in adolescents with T1DM and reshaping illness perception. These findings underscore the importance of integrated psychological support to improve adjustment and quality of life in chronic illness during adolescence.

## 1. Introduction

Type 1 diabetes mellitus (T1DM) is one of the most prevalent chronic diseases around the world [1,2,3]. The onset of this condition typically occurs during childhood and adolescence [4], although it can also manifest in adulthood [5]. The disease is primarily characterized by destruction of the *β*-cells within the pancreas [6], consequently necessitating continuous insulin administration, rigorous blood glucose control, and a controlled diet. The prevalence of the disease has increased steadily from 200 million cases in 1990 to 830 million in 2022 [7]. In 2022, approximately 8.75 million individuals worldwide were living with T1DM, including 1.52 million (17%) under the age of 20, with an estimated 182 thousand deaths [8].

The incidence of T1DM peaks during adolescence [9], a stage in which the management of the condition can be complicated due to the presence of age-specific characteristics [10]. From a physiological perspective, puberty is accompanied by a series of hormonal alterations, culminating in heightened insulin resistance and a concomitant decline in metabolic control [11]. Concurrently, this developmental period is characterized by an intrinsic inclination towards exploration and the pursuit of novel experiences, accompanied by a marked tendency towards autonomy from parental figures and a propensity to establish connections with peers. These factors have the potential to influence an individual’s ability to adapt and adjust to the disease in an optimal manner [12]. The physiological, social, and psychological aspects of living with T1DM are challenging [13]. A plethora of self-care guidelines have been identified, including continuous glucose monitoring, insulin administration, adherence to a prescribed diet, and consistency with physical exercise [14,15], and could have the potential to contribute to added stress [16]. These tasks are frequently assigned to primary caregivers during the early years of an individual’s life. Nonetheless, as adolescents progress through the maturation process, they progressively assume a greater degree of responsibility for their self-care [17]. This transition may have an impact on the well-being and quality of life of these adolescents [18] and may extend into adulthood [19,20]. In adolescents diagnosed with T1DM, higher HbA1c levels have been observed to be associated with diminished health-related quality of life [21].

In this regard, adolescents with T1DM exhibit higher levels of anxious-depressive symptoms and behavioral problems compared to their peers without the disease [22,23]. These psychosocial issues have the potential to hinder effective disease management and treatment adherence [24]. In the study conducted by Iina et al. [25], a negative reciprocal relationship was observed between depressive and anxious symptoms and glycemic control. Specifically, an increase in the presence of depressive symptoms was found to have a negative impact on glycemic control, while the presence of anxiety constituted a risk factor for the onset of depression and anxiety [25]. A similar correlation has been observed between quality of life and glycosylated hemoglobin (HbA1c) levels. In this regard, higher HbA1c levels (indicative of poorer glycemic control) have been associated with lower quality of life [26]. The prevalence of eating disorders and substance abuse has also been demonstrated to be higher among this adolescent population compared to their peers without the disease [27,28].

Given the pivotal role that peers play in the lives of adolescents with T1DM, social relationships have the potential to exert a significant, either positive or negative, influence on their disease management [29]. Many of these adolescents encounter difficulties in disclosing their disease-related concerns due to the fear of being rejected or socially isolated, which can ultimately result in a lack of acceptance and integration into their daily lives regarding their chronic condition [23]. The search for identity in these adolescents is closely related to this aspect of their lives [30], where self-esteem plays a pivotal role in the formation of self-concept. These constructs have been linked to the management of T1DM, particularly to glycemic control. This suggests that monitoring these associated variables may be more crucial than behavioral aspects in improving diabetes-related outcomes [31].

Regarding the influence of family dynamics in coping with T1DM, strong family cohesion and parental support are consistently associated with better metabolic control and psychological adjustment. In a cross-sectional study, higher family cohesion correlated significantly with lower HbA1c levels in adolescents, suggesting that supportive home environments facilitate self-management and communication between adolescents and caregivers [32]. Conversely, excessive parental control or conflict have been identified as detrimental to adolescents’ autonomy and mental well-being, slowing the transition to effective self-management [33].

In recent years, several psychoeducational programs have been developed with the aim of improving both clinical outcomes and psychological adjustment in adolescents with T1DM. Evidence from systematic reviews, including the work of Luque et al. [34], suggest that, although these interventions may not consistently translate into significant changes in objective metabolic indicators like glycated hemoglobin (HbA1c), their contribution should not be underestimated. Some of these programs have demonstrated substantial benefits in enhancing adolescents’ quality of life by reducing emotional distress, promoting adaptive coping strategies, strengthening self-esteem, and improving illness perception [35]. By targeting socioemotional factors that directly influence treatment adherence and day-to-day disease management, these interventions can foster healthier adjustment processes during a critical developmental stage, ultimately equipping adolescents with the psychological resources necessary to navigate both the immediate and long-term challenges of living with a chronic condition [36].

Following a comprehensive review of the most recent interventions which try to improve quality of life in adolescents with T1DM as well as promote a correct adjustment to this disease, both in-person and digital, it was observed that most of those developed to date lack a solid and clearly defined theoretical basis, as well as a consensus on the theoretical constructs on which they are based. It is evident that the fundamental focus of these programs is predominantly oriented towards psychoeducational aspects of the disease and enhancing therapeutic adherence. However, there is a conspicuous absence of incorporation of other indicators that are of paramount importance for the enhancement of the well-being of these patients—physical well-being, cognitive coping, emotional well-being, social relationships and support, and identity [37].

In light of the aforementioned lack of theoretical consistency in extant intervention programs, the 10VIDA intervention was designed and developed. The proposal is a specific psychological intervention for T1DM adolescent patients, focusing on psychoeducational and socioemotional aspects. This program provides tools to improve coping with the disease and psychological well-being, throughout seven sessions, of which five are individual sessions aimed at adolescents with T1DM and two include primary caregivers. Its delivery may be conducted in a blended format, offering both in-person and online attendance, with the choice of modality determined by the preferences of the enrolled participants. The focus of these sessions is on adjustment to the disease, self-esteem and self-concept, coping strategies, emotional regulation, social issues, the needs of caregivers, and the family system [38].

A salient and pioneering element of this program is its foundation in the Disease Adjustment Model from an Integrative Perspective (DAMIP) [39]. This theoretical framework synthesizes concepts from a range of extant theoretical models, leveraging their complementarity. Based on Livneh’s Integrative Model [40,41], it also incorporates elements of Antonovsky’s Salutogenic Model [42], Leventhal’s Self-Regulation Model of Illness [43], and the Health Belief Model (HBM) [44]. Furthermore, five quality-of-life indicators were identified from the extant scientific literature: physical well-being, cognitive coping, emotional well-being, social relationships and support network, and identity [39].

Following these considerations, the 10VIDA program was devised with the objective of addressing a range of psychological goals among the adolescent population, with a particular focus on promoting coping and socioemotional management of T1DM during the adolescent years. The intervention comprises five sessions, with the primary objective of facilitating adjustment to the disease, along with physical and mental health. This is achieved through the provision of psychosocioemotional tools [38].

The objective of the present study was to examine the psychosocioemotional characteristics associated with adjustment to the disease and psychological well-being in the adolescent population with T1DM, with a particular focus on the potential benefits of the 10VIDA program on the health of these adolescents.

In relation to the aforementioned objective, the following hypotheses are proposed: (1) There will be notable discrepancies in the primary psychological variables examined according to gender and the presence of an additional disease besides T1DM, with a more adverse psychological state being anticipated in girls and in those individuals living with an additional disease; (2) the 10VIDA program will result in a substantial enhancement in general well-being, as well as a reduction in psychopathology and general malaise, which will be examined in two ways: (a) at the intrasubject level, the change in the measures of a single sample will be observed at three time points: 6 months before the commencement of treatment, immediately prior to the commencement of treatment, and 6 months following the conclusion of treatment; (b) at the intersubject level, a comparison will be made between the experimental group (which received the 10VIDA program) and the waiting list control group. It is anticipated that the adolescents in the experimental group will demonstrate superior indicators and a healthier profile.

## 2. Materials and Methods

### 2.1. Description of the Sample

To qualify for inclusion in this study, participants were required to meet the following criteria: (a) be between 12 and 16 years of age, (b) have suffered from T1DM for at least 6 months, and (c) regularly attend the pediatric endocrinology outpatient service of a hospital for medical follow-up. Patients suffering from the following conditions were excluded from participation in this study: (a) infantile cerebral palsy (ICP) or epilepsy, (b) brain tumors, (c) attention deficit or hyperactivity disorder (ADHD), (d) mental retardation, (e) genetically determined syndromes, and (f) any psychological diagnosis prior to the onset of the diagnosis of organic disease.

All patients were recruited at the Hospital General Universitario in Valencia, Spain, a reference center in pediatric endocrinology. Subsequent to the establishment of these criteria, the research initially included a sample of 19 adolescents. Two studies were derived from this sample, which are analyzed in the following sections.

The present study was conducted as a pilot study, with acknowledged limitations in terms of statistical power (e.g., the sample size was not calculated a priori based on an assumed effect size, significance level (α), and statistical power (1-β)).

### 2.2. Design and Procedure

In the present study, we present the findings of two empirical investigations. The initial study was an intersubject longitudinal investigation comprising a waiting list control group and an experimental group. The control group, consisting of participants who were part of the waiting list control group (n = 9), underwent evaluation at two time points, with an interval of six months between them. During this period, the subjects did not receive any form of psychological intervention. The experimental group (n = 10) was also evaluated on two occasions: the first, immediately prior to the commencement of the 10VIDA intervention, and the second, six months following the conclusion of the program. To ensure that the two groups were initially comparable, an inter-subject analysis was conducted at the initial evaluation time point (T1, pre-treatment) to confirm the absence of significant differences in the variables under study. Subsequently, at the second evaluation time (T2), an intersubject analysis was conducted to examine the differences between the groups and to analyze the benefits of having received or not received the 10VIDA intervention.

The allocation sequence was generated using a computerized random number generator. A simple randomization procedure was applied, based on a single series of random assignments. Participants were allocated to the intervention groups (experimental and control) using the 4.5.0 version of software R, ensuring that each participant had an equal probability of being assigned to either group.

To maintain allocation concealment and minimize the risk of selection bias, the generation and management of the sequence were conducted in a controlled and secure setting. The sequence produced by R was stored in such a manner that precluded the principal investigators from accessing the allocation information prior to the enrollment of the participants. The assignment process was executed by an independent researcher external to the study team, who was responsible for direct interaction with the participants, thereby facilitating the maintenance of blinding.

The integrity of the procedure was periodically monitored to prevent any potential manipulation of the assignments. Thus, both participants and the investigators responsible for the intervention remained unaware of the allocated groups, which minimized bias and strengthened the internal validity of the results.

The second study was an intrasubject longitudinal analysis comprising a sample of 13 adolescents with T1DM, who were evaluated at three distinct time points. The time points were as follows: a first pre-treatment assessment (T1), another pre-treatment assessment at 6 months (T2), and finally, a new assessment (T3) carried out just after the 10VIDA intervention program, which spanned six months, had been completed. The treatment was conducted over the course of five individual one-hour sessions, with a monthly frequency. The remaining month was allocated to the 15-day interval between the T1 evaluation and the commencement of the treatment period, as well as the 15-day interval preceding the post-treatment evaluation.

The 10VIDA program is a multifaceted initiative that offers strategies and tools designed to address the needs of adolescents. These initiatives are intended to enhance adherence to treatment regimens, thereby facilitating improvements in both physical and psychological health outcomes.

The precise aims of each adolescent session, along with the specific areas to be addressed, are enumerated in Table 1.

To that end, a series of activities and tasks have been developed to facilitate the achievement of the aforementioned objectives. These activities and tasks have been meticulously designed to enhance each of the fundamental domains of the sessions. The following activities have been designed for this purpose:

S1. My beliefs. The activities to develop are:

(a) Well-lived chronic disease: Provide the adolescent a leading role and create a safe space for them to express themselves and grow emotionally. Recognize the main concerns that generate fear and the place of their illness within them.
(b) Deal: Express and recognize feelings, beliefs, and thoughts about their illness. To provide tools for decision making in their life beyond the disease. Respond to their emotions and thoughts to guide them towards achieving their goals and coping with their fears.

S2. A look inside me. The activities to develop are:

(a) I am and not my disease: Work on the importance of self-reflection. Encourage learning to look at and discover oneself with serenity and affection. Define self-esteem and self-concept. Reflect on the role of the disease in one’s self-image.
(b) Because I’m worth it: Self-awareness and self-discovery: strengths, weaknesses, and needs.
(c) I spoke nicely: Reflect on the importance of words and their effect on thoughts and action. Work on positive language and the effect of negative thoughts.

S3. From serenity. The activities to develop are:

(a) I listen to my signs: Through an explanation supported by drawings, learn the difference between fear and anxiety. Understand how fear/anxiety works. Learn how to detect and identify signs of fear/anxiety in your body.
(b) My monster and I: Give name, form, “life” to the “monster” to have more control and stop fearing it. Training in self-regulation resources.
(c) The eye outwards: Explain the mindfulness-based technique: mindfulness in everyday life. Learn to discover and be amazed by their surroundings and what they may not have noticed.

S4. Emotions: My Friends. The activities to develop are:

(a) Emotions, my friends: Define the concept of emotions, their functions, and their meanings.
(b) Emociometer: Continue working with emotions and their functions. Increase emotional vocabulary. Training in emotional skills.
(c) D&D: Discover effective strategies for expressing emotions.

S5. A look outside. The activities to develop are:

(a) I’m not alone: Understanding the influence of others in our lives. To observe how the disease has affected relationships and social situations.
(b) Social superhero: Demonstrate the main social skills for their stage of development and describe the skills with them social skills training.
(c) Color glasses: Learning to perceive the world through a more positive language.

Each subject was informed about the objectives and methodology of this study and provided with written consent to participate. The specialist physician obtained the requisite consent and assent. The data were treated with confidentiality and anonymization and were utilized exclusively for the purposes of this study, in accordance with the Data Protection Act of 27 April 2016 (GDPR). A numeric code was employed to link the identifying information of each participant. The data were stored in a locked cabinet at the principal investigator’s place of work, and the electronic data were password-protected on the university network computer. Any modifications to the protocol were duly recorded on ClinicalTrials.gov (NCT04476433).

This study was conducted in accordance with the ethical guidelines set forth in the 2013 World Medical Association Declaration of Helsinki and was granted approval by the Human Research Ethics Committee of the Universitat de València (Reference: 1226194) and the Hospital General Universitario de Valencia (Reference: 151/2021). Appropriate measures were taken to guarantee the total confidentiality of the participants’ data, in accordance with the Organic Law on the Protection of Personal Data (LOPD) 3/2018, of 5 December.

### 2.3. Analyzed Variables

A computerized questionnaire was developed using the measurement instruments described below and implemented through LimeSurvey, an online evaluation platform provided by the University of Valencia.

#### 2.3.1. Sociodemographic Variables

The sociodemographic variables of gender, age, and family socioeconomic level of the adolescents were collected through an ad hoc registry. Furthermore, clinical variables were documented, encompassing the primary CD diagnosis, any secondary diagnoses, the time of diagnosis, the time of treatment, the number of hospital and/or emergency room admissions, and the total number of these admissions directly related to CD. In addition, the type and amount of medication taken, as well as the frequency of regular medical control visits, was meticulously documented.

#### 2.3.2. Psychological Variables

**The Rosenberg Self-Esteem Scale (RSE)** [45]. This questionnaire was employed to assess self-esteem. The Rosenberg Self-Esteem Scale (RSE) was employed for the assessment of self-esteem in adolescents with DM1. Specifically, the Spanish translation utilized by Atienza et al. [46] was employed. This instrument is employed for the assessment of self-esteem in adolescents with DM1. This scale is among the most widely utilized for the global measurement of self-esteem and is composed of 10 Likert-type items (scale from 1—Completely disagree to 4—Completely agree). The content of this questionnaire is focused on feelings of self-respect and self-acceptance and is comprised of five positively and five negatively worded items. The scale has demonstrated satisfactory psychometric properties, as evidenced by a reliability coefficient of 0.86 in the Atienza et al. [46] study. The reliability of the questionnaire was 0.84 for this study.

**The Psychological Well-Being Scale for Adolescents (BIEPS-J)** [47] was utilized to assess the psychological well-being of the subjects. The present scale is based on Ryff’s [48] Multidimensional Model of Psychological Well-Being. The questionnaire is composed of 13 items based on the Likert scale, with three response options (1 = disagree, 3 = agree) distributed in four subscales: control of situations, which refers to feelings of control and self-competence; psychosocial bonds, which encompass warm and trusting relationships with others; self-acceptance, which entails accepting one’s physical and psychological characteristics; and projects, which refer to the presence of life goals and projects that imbue one’s life with meaning. The assessment tool provides the capability to derive a score for each of the subscales, as well as an overall score for psychological well-being by calculating the mean of the scores for each of the subscales. In terms of reliability, the original study yielded a reliability value of 0.74 for the total questionnaire, 0.56 for situational control, 0.51 for prosocial bonds, 0.55 for projects, and 0.50 for the subscale of self-acceptance. In regard to the present study, the reliability coefficients obtained for the respective scales were as follows: 0.54 for the “control of situations” scale; 0.54 for the “prosocial bonds” scale; 0.76 for the “personal projects” scale; 0.72 for self-acceptance; and an alpha of 0.74 for the total scale.

**Strengths and Difficulties Questionnaire (SDQ)** [49]. The purpose of this questionnaire is to evaluate the potential psychopathology and emotional adjustment of adolescents with DM1. The questionnaire’s primary objective is to identify potential emotional and behavioral disorders in children and adolescents between the ages of 4 and 16. The questionnaire contains 25 Likert-type items, with three response options (0 = not true, 2 = truly yes), distributed across five subscales: emotional symptomatology, behavioral problems, hyperactivity, problems with peers, and prosocial behavior. A score can be obtained for each of the aforementioned subscales, in addition to an overall score for the questionnaire. This calculation is derived by summing the scores from the remaining subscales with the exception of the subscale designated as “prosocial behavior.” The questionnaire has been extensively used in various studies, with previous investigations involving individuals with chronic respiratory disease demonstrating its satisfactory reliability. For example, Valero-Moreno et al. [50] reported reliability coefficients of 0.72 for the total scale, 0.61 for emotional symptoms, 0.43 for behavioral problems, 0.63 for hyperactivity, 0.49 for prosocial behavior, and 0.61 for problems with peers. In our study, the reliability coefficients for each scale were as follows: an alpha of 0.770 was obtained for the “emotional symptoms” scale, 0.640 for “behavioral problems,” 0.617 for “hyperactivity,” 0.692 for “peer problems,” 0.660 for “prosocial behavior,” and finally, 0.824 for the overall scale.

**Hospital Anxiety and Depression Scale (HADS)** [51]. This instrument, originally developed by Zigmond and Snaith [51], is employed for the assessment of cognitive clinical features associated with anxiety and depression in adolescent samples. In the case of adolescents, the version adapted and validated by Valero-Moreno et al. [52] was utilized. This version consists of 11 items (compared to the original 14) on a Likert-type scale with a four-point range from 0 to 3. As in the original version, it is composed of two subscales, namely “depression” and “anxiousness.” In the present study, the questionnaire demonstrated a reliability index of 0.83 for the anxiety factor in adolescents, an alpha of 0.79 for the depression factor, and a reliability index of 0.89 for general emotional distress in the adolescent sample.

**Parent’s educational style evaluation scale (EP)** [53]. The present study employed a questionnaire designed to assess children’s perceptions of their parents’ educational style. The questionnaire endeavors to categorize a series of behaviors and attitudes exhibited by the child’s parents along a number of dimensions, which are grouped into six distinct subscales. The subscales are as follows: affection and communication (eight items), autonomy promotion (eight items), behavioral control (six items), psychological control (eight items), disclosure (five items), and humor (six items). Accordingly, the comprehensive questionnaire comprises 41 Likert-type items with five response options, ranging from 1 (strongly disagree) to 6 (strongly agree). The questionnaire demonstrates satisfactory reliability for all subscales in analogous samples. The reliability coefficients for the aforementioned subscales were as follows: 0.92 for affect and communication, 0.88 for autonomy promotion, 0.82 for behavioral control, 0.86 for psychological control, 0.85 for disclosure, and 0.88 for mood [53]. In this study, the reliability indices for the subscales were as follows: 0.952 for “affection and communication,” 0.845 for “autonomy promotion,” 0.827 for “behavioral control,” 0.813 for “psychological control,” 0.864 for disclosure, and 0.848 for “humor.”

**Emotional Skills and Competencies Questionnaire (ESCQ-21)** [54]. This questionnaire was used to assess the socioemotional competencies of adolescents with DM1. To this end, a questionnaire was administered to the participants. Specifically, a reduced and validated version of the scale developed by Takšić et al. [54] was used in the adolescent population, which was subsequently adapted to Spanish [55,56]. The Emotional Competence Scale for Children and Adolescents (ESCC-21) is a 21-item instrument comprising three subscales that assess different dimensions of emotional competencies. The first subscale, Perceiving and Understanding Emotions (PC), assesses an individual’s capacity to discern and differentiate emotions in themselves and others. The remaining subscale, “Managing and regulating emotions (MR),” evaluates an individual’s capacity to regulate emotions effectively, adjusting them to obtain desired outcomes. The instrument has demonstrated satisfactory psychometric properties, as evidenced by the study conducted by Schoeps et al. [56], which yielded an alpha value of 0.84 for the PC scale, an alpha of 0.90 for EE, and an alpha of 0.74 for MR. In our case, the Cronbach’s alpha values obtained were 0.81 for PC, 0.96 for EE, and 0.87 for MR.

**Brief Illness Perception Questionnaire (B-IPQ)** [57]. To assess the cognitive and emotional representations of adolescents with DM1 regarding their disease, this scale developed by Broadbent et al. [57] was employed. Specifically, this study utilized a version of the scale that has been adapted and validated for pediatric chronic disease [50]. This scale consists of 8 Likert-type items (0 to 10) and a final open-ended item that asks the subject about the etiology that the patient attributes to his or her disease. Each of the items of the scale represents a distinct dimension related to the disease, including the following: identity, consequences, duration, personal control, control of treatment, worry, understanding, and emotional response. The total score of illness as a perceived threat to the subject is obtained by adding the responses given to these items. In terms of its internal consistency, this instrument has exhibited adequate predictive and discriminant validity [57], along with a Cronbach’s alpha of 0.80 in a Spanish sample with chronic illness [58]. In the present study, the Cronbach’s alpha value obtained was 0.503.

#### 2.3.3. Statistical Analysis

All statistical analyses were conducted using the statistical software program “SPSS Statistics,” version 28.0.1.1, on the Windows 11 operating system. In order to respond to the aforementioned hypotheses, it was necessary to perform an analysis of the most relevant descriptive statistics (mean, median, standard deviations, etc.), a normality analysis (Shapiro–Wilk), and a reliability analysis (Cronbach’s alpha). Alpha, inter-, and intra-group comparisons of means were conducted, employing both parametric and non-parametric tests (Student’s T, Mann–Whitney U, and Friedman). Furthermore, the effect size of these comparisons was measured using Cohen’s d and r.

## 3. Results

### 3.1. Sociodemographic and Psychological Variables of Adolescents

As illustrated in Table S1 the initial clinical and sociodemographic characteristics of the adolescents diagnosed with T1DM were delineated. The sample population comprised 11 males (57.9%) and 8 females (42.1%). The majority of these adolescents did not present with any secondary disease or psychological disorder (63.2%), and the majority also did not require the administration of adrenaline (94.7%). With regard to the frequency of visits to the specialist, in this case, the endocrinologist, the majority of adolescents (73.7%) visited the specialist every three months.

The subsequent phase involved the acquisition of the profile of the primary quantitative variables of this study for the adolescents with T1DM (Table S2). The age of the subjects ranged from 11 to 17 years, with a mean of 13.31 years (standard deviation = 1.66). With regard to the clinical variables associated with the primary disease, the mean duration of treatment was observed to be 43.21 months (range = 5–138). During the course of their treatment, the adolescents with T1DM in the sample exhibited a low incidence of hospital admissions due to T1DM (M = 1.68; median = 2) and a correspondingly low duration of hospitalization (M = 1.05 days).

With regard to the psychological and family variables, the adolescents exhibited a mean self-esteem, as measured through the RSE, that could be classified as medium-high (M = 30.52; SD = 6.01). This is predicated on the premise that the scores can fluctuate from 0 to 40. Furthermore, the BIEPS results indicated a psychological well-being. The mean score was 34.84 (SD = 3.37), which is at the scale’s midpoint (max = 66). However, the “projects” subscale demonstrated a higher mean score (M = 7.47, SD = 1.67), given that the maximum attainable score on this subscale is 36. The median score has been established at 8.

The mean scores obtained through the HADS instrument indicate higher levels of anxiety (M = 4.26, SD = 3.46) than depression (M = 2.26, SD = 2.20). However, both scores fall below the cutoff points for pathological cases (6 and 5.4, respectively).

Conversely, the scores obtained in the subscales of the SDQ are within the normal range according to the scale norms, with “peer relations” (M = 2.26, SD = 2.25) and “behavioral problems” (M = 2.15, SD = 1.89) being the subscales most closely aligned with their respective cut-off points (3).

With regard to the perceived parenting styles, the high scores obtained in the scales of “affection and communication” (M = 41.36, SD = 8.96) and “promotion of autonomy” (M = 40.68, SD = 5.70) are noteworthy. This finding suggests that adolescents may be perceiving a parenting style that endorses autonomy within established constraints, fostering an environment conducive to open communication with their parents.

However, the scores on the “psychological control” subscale could also be considered to be relatively high. The mean score observed was 22.63 and the median was 24 (SD = 8.15), suggesting that these values are relatively close to the maximum score of 30 on the scale. This may indicate that adolescents perceive that their caregivers occasionally employ some strategies of a negative nature, such as emotional blackmail.

The mean scores on the ESCQ subscales are highly similar, and, given that the maximum score that can be reached on them is 6, it can be stated that the adolescents in our study possess average emotional competencies, with their emotional perception and understanding being particularly noteworthy (M = 4.74, SD = 0.675).

The mean score on the B-IPQ was 32.79, which can be classified as medium-low. This finding indicates that adolescents in our study do not perceive the disease associated with type 1 diabetes mellitus as a substantial threat.

### 3.2. Main Correlations

As illustrated in Table S3, the primary variables under investigation exhibit a range of correlative relationships. Given the substantial number of significant correlations, the present commentary will be constrained to the most pertinent ones, namely those that do not occur between subdimensions of the same questionnaire and that are also high (r > 0.50).

Among the variables associated with adolescents with T1DM, it is notable that the “emotion management and regulation” dimension (ESCQ) demonstrates a positive and significant correlation with the “control of situations” dimension (ρ = 0.541) and with time in treatment (ρ = 0.503). This finding suggests that adolescents who have been in treatment for a longer period and who are able to adaptively regulate their emotional responses may perceive a greater sense of control over the situations occurring in their environment.

In addition, the general emotional well-being of these adolescents exhibits a positive and statistically significant correlation with the “autonomy-promoting” parenting style (ρ = 0.740) and a negative correlation with the potential presence of psychopathology (SDQ) (ρ = −0.706). This parenting style, which fosters the development of children’s independent thinking, has been found to be positively associated with a heightened sense of control over situations experienced by the adolescent (ρ = 0.668) and an enhanced self-acceptance (ρ = 0.608). Furthermore, a negative correlation was observed between general emotional distress and total perceived well-being (ρ = −0.568 *) as well as the domain of acceptance (ρ = −0.729 **). The adequate expression of emotions has been demonstrated to be positively correlated with both acceptance and overall adolescent well-being (ρ = 0.792 **) and negatively correlated with overall emotional distress (ρ = −0.750 **). The parental style of “Affection and Communication” has also been demonstrated to exhibit a positive correlation with adolescent well-being (ρ = 0.568) and a heightened perception of life projects among adolescents (ρ = 0.598).

### 3.3. Comparisons of Pre-Intervention Groups

Subsequently, a comparison was conducted between the means according to gender and the presence of additional diseases or disorders in addition to type 1 diabetes mellitus. Prior to conducting the aforementioned analyses, it was necessary to ascertain whether the distributions of the variables across the various groups exhibited a normal curve. This objective was accomplished by employing the Shapiro–Wilk statistic and supplementary graphical instruments, including histograms.

Consequently, it was determined that not all variables exhibited a normal distribution. Consequently, both parametric and nonparametric tests were conducted, depending on whether the variables met or did not meet the criteria for normality in their distribution. We used Student’s *t*-test to compare the variables that met the assumption of normality. Conversely, for variables that did not meet this assumption, the nonparametric alternative, the Mann–Whitney U test, was utilized. As the nonparametric test employs the median statistic for comparison between the two samples, as opposed to the mean (as is the case with the Student’s *t*-test), we made two different tables displaying the meaningful values in each case.

The effect size was also articulated through a range of statistical metrics, contingent upon the particulars of the analytical investigation. For instance, Cohen’s d was utilized for the *t*-test, whereas the “r” statistic was employed for the Mann–Whitney and Friedman U tests.

### 3.4. Comparison According to Gender

Firstly, a comparison was conducted according to the gender of the adolescents, the results of which are presented in Tables S4 and S5. The findings indicate that boys demonstrated significantly higher scores in emotional well-being and self-esteem (*p* < 0.05), while girls exhibited higher scores in emotional distress and clinical anxiety (*p* < 0.01), depression (*p* < 0.05), and illness perception (*p* < 0.1). The data shows a tendency among girls to perceive T1DM as a greater threat to their health than boys and to exhibit significantly higher levels of emotional distress. Furthermore, the findings underscore that male subjects exhibit superior skills in emotion management and regulation, with significantly better performance than girls in expressing and labeling emotions (*p* < 0.01). However, no gender differences were observed in the perception and understanding of emotions.

#### 3.4.1. Comparison According to the Presence of Comorbidities

Tables S6 and S7 present a comparison of the most pertinent variables in relation to the presence of an additional disease or psychological disorder in conjunction with T1DM. No significant disparities were observed in the dimensions of general well-being as perceived by the adolescents, nor in their perceived levels of anxiety or depression. Conversely, adolescents afflicted with an additional disease did not demonstrate a noticeably elevated perception of illness in comparison to those with exclusively T1DM.

#### 3.4.2. Comparison Between Control and Experimental Groups

A comparison of the pretreatment means was conducted using the Mann–Whitney U test and Student’s *t*-test, as appropriate, to ascertain whether there were significant differences between the experimental and control groups prior to the intervention.

Table 2 and Table 3 illustrate the most notable discrepancies in the scores of the variables under investigation when comparing the means of the control and experimental groups at the second evaluation point (after the experimental group had received psychological treatment). In general, it can be observed that a considerable number of the obtained scores are not statistically significant. However, an examination of the direct data reveals a tendency towards improvement in the majority of the aspects treated therapeutically in the adolescents through the 10VIDA protocol.

A review of the scores obtained by adolescents with T1DM in self-esteem, emotional well-being (both in general and in aspects related to control of situations, bonds, projects, and acceptance), and emotional skills and competences (whether in “Perceiving and understanding emotions (PC),” “Expressing and labeling emotions (EE),” or “Managing and regulating emotions (MR)”) reveals slightly higher mean values in the experimental group than in the control group. Conversely, the scores for anxiety, depression, and general clinical emotional discomfort, as well as problems with peers and perceived threat of illness, were found to be lower in the group that received treatment. However, no significant improvement was observed in scores related to conduct problems, hyperactivity, or prosocial behavior among the adolescents who received treatment.

An examination of the perception of adolescents with T1DM regarding the educational style of their primary family caregivers reveals that the experimental group exhibited higher scores in autonomy promotion, behavioral control, and psychological control. However, the scores associated with affect and communication, autonomy promotion, disclosure, and humor did not demonstrate a statistically significant change following the psychotherapeutic process.

### 3.5. Comparison of the Means of the Intrasubject Longitudinal Study

As illustrated in Table 4, the Friedman test was employed to compare the various study variables at distinct time points. This table depicts the change observed in adolescents with T1DM who had not received the life protocol (change to be observed in the scores between times 1 and 2 of evaluation) and after receiving psychological treatment (change between times 2 and 3 of evaluation). Due to the substantial volume of data being examined, the table presents only the most salient information, whether due to its statistical significance or trend, or the importance of elucidating the meaning of the scores, even if they were not statistically significant.

A thorough examination of the data revealed that the majority of the results were not statistically significant. However, the trend did indicate positive therapeutic changes in the adolescents following the administration of the 10VIDA treatment.

A global analysis of the well-being scores reveals an initial decline over time in the absence of treatment, followed by a statistically significant enhancement following the implementation of the 10VIDA therapeutic intervention. This phenomenon is manifest in the total score for well-being, as well as in all the specific aspects of well-being related to control of situations, bonds, projects, and acceptance.

With regard to psychopathological aspects, the data indicate a notable increase in anxiety, depression, general emotional discomfort, and peer relationship issues among adolescents who were not exposed to the 10VIDA program (t1 and t2 comparisons). Conversely, a substantial enhancement was observed, accompanied by a significant decrease in these variables. The values of this psychological clinic after the subjects have undergone the treatment program (comparing t3 with the remaining evaluation times) also demonstrate improvement in terms of prosocial behavior and adolescents’ socioemotional skills and competence (perception and understanding, expression and labeling, management and regulation).

However, the elements associated with self-esteem, illness perception, behavioral issues, and hyperactivity do not appear to have been significantly enhanced by the adolescents’ participation in the 10VIDA program. The data do not demonstrate a discernible impact of the intervention on the aforementioned indicators.

## 4. Discussion

Type 1 diabetes mellitus is the most prevalent chronic disease in the pediatric population, and it presents a series of challenges in addition to those inherent to the adolescent stage. Scientific evidence indicates that these adolescents are particularly vulnerable to psychological problems and disorders, which can negatively impact disease management and coping, as well as the quality of life of these individuals.

With respect to the initial hypothesis, the present study validated the proposed approach by disclosing substantial disparities in various psychological variables according to gender. Regarding general well-being, male subjects exhibited superior outcomes in comparison to their female counterparts. Specifically, female subjects demonstrated elevated levels of depressive and anxiety symptoms, with the latter exhibiting a marked increase over that observed in male subjects. Furthermore, girls exhibited heightened emotional symptoms, with statistically significant scores observed on this subscale and on the total SDQ questionnaire score. Conversely, the prevalence of illness perceptions was found to be considerably higher among female subjects compared to their male counterparts. These findings align with those reported by Ouzouni et al. [23], who documented a higher prevalence of anxiety-depressive symptoms and low self-esteem in girls. These authors underscored the elevated risk of depression among adolescents with T1DM in comparison to their peers without the disease, a factor that may ultimately impact disease management and adaptation. Concurrently, Harrington et al. [25] identified significant disparities in depressive symptoms based on gender, with elevated rates observed among female subjects. However, since other studies, such as the one conducted by Hadad et al. [22], have demonstrated a higher incidence of emotional problems among boys, while behavioral problems and hyperactivity were more prominent among girls, further research is necessary to more thoroughly examine these disparities.

In regard to the construct of self-esteem, the present study reveals statistically significant disparities based on gender, with male subjects demonstrating comparatively higher mean scores. These results are consistent with those described in other studies, such as the one conducted by Kenowitz et al. [31], who, despite not finding significant differences, observed a tendency among girls to report lower self-esteem compared to boys. Furthermore, the investigation delved into the relationship between this variable and the format of insulin administration, leading to the discovery of a positive association between elevated levels of self-esteem and the preference for insulin pumps over injections as a mode of insulin administration.

It is noteworthy that the present study did not reveal a correlation between the age of the participants and the psychological variables under consideration. In this regard, the results presented in other similar studies vary; while some of them have reported an association between age and emotional and behavioral problems [22], others have found no such relationship [31]. These discrepancies could be attributed to two factors. First, there is the gradual familiarization with the disease and its treatment over time. And second, there is the perceived social and family support among the adolescents in our sample.

Conversely, glycemic control and HbA1c levels, despite not being psychological variables, are nevertheless associated with the latter. For instance, in the aforementioned study by Harrington et al. [25], an inverse relationship between the severity of depressive symptoms and glycemic control was identified. Concurrently, evidence has emerged indicating that ineffective diabetes management is associated with an elevated prevalence of behavioral and emotional challenges among this demographic. However, the present study did not reveal such an association.

Contrary to the findings of the preceding approach, the present investigation did not substantiate the second one for the first hypothesis, since no significant discrepancies were observed in the prevalence of anxiety and depressive symptoms among the adolescents with T1DM, irrespective of the presence of concomitant diseases or the duration of treatment. Additionally, these adolescents did not exhibit a significantly heightened perception of the disease in comparison to those who did not have comorbid T1DM.

As previously indicated, there are currently various psychological interventions for adolescents with T1DM that focus primarily on aspects of diabetes education; however, none of them include other psychological variables that are highly relevant to the quality of life and overall well-being of these adolescents [37]. The integration of these novel indicators has the potential to yield not only psychological enhancement but also, indirectly, physical improvement. The 10VIDA program was developed as a tool to address the psychosocial and emotional aspects of adolescents with T1DM based on the relationship between physical and psychological well-being [34], hypothesizing that it would have a positive secondary impact on medical variables such as HbA1c levels or glycemic control. The intervention program under consideration was designed based on the DAMIP Model [39], whose integrative style allows for the consideration of all quality-of-life indicators identified in the existing scientific literature.

Consequently, consistent with the second hypothesis concerning the efficacy of the 10VIDA intervention program, the findings of this study demonstrate a conspicuous downward trend in the scores obtained on psychological variables such as general malaise, anxiety-depressive symptoms, and perceived threat of illness within the experimental group, in contrast to the control group. Conversely, an additional positive trend is observed in the scores obtained in the experimental group in variables such as psychological well-being and self-esteem.

A similar phenomenon can be observed in the intrasubject study, where there is evidence that adolescents’ scores for general well-being showed an upward trajectory after the intervention. Concurrently, a significant decline in the general distress variable was observed post-treatment.

The present study acknowledges several limitations that should be considered when interpreting the findings. Firstly, one of the main limitations of the present study is the relatively small sample size of both the intervention and the control groups. Although this constraint reduces the statistical power and generalizability of the findings, this study was intentionally designed as a preliminary pilot investigation. Future studies should justify sample sizes more explicitly, taking into account effect size assumptions and feasibility, and should recruit larger and more diverse populations to enhance statistical power and generalizability. Secondly, the use of a waitlist control group may have introduced expectation effects and lacked blinding, which could bias outcomes. Alternative approaches, such as active comparator interventions or cluster randomization, should be considered in future trials to minimize bias. Thirdly, the limited follow-up period restricted the evaluation of long-term effects. The scientific literature, with interventions such as that of Jaser et al. [59] and systematic reviews such as that of Charalampopoulos et al. [60], underscores the significance of intervention duration, which can be a pivotal factor in demonstrating a notable enhancement in emotional well-being. In this regard, our study provides a framework for integrating change over a six-month period, facilitating the acquisition and consolidation of knowledge. We emphasize the necessity of continuing to contrast the benefits of receiving the 10VIDA program in larger samples and with more post-treatment follow-up measures. Longer follow-ups (6–12 months) are needed to assess sustainability. Fourthly, recruitment from a single region may limit representativeness, underscoring the importance of broader sampling strategies. In addition, intervention fidelity was not systematically monitored; future studies should include facilitator training, adherence checks, and quality control procedures to ensure delivery consistency.

Furthermore, because 10VIDA integrates psychoeducation, behavioral regulation, and socioemotional skills training, it remains unclear which components drive the effects; dismantling studies could clarify these mechanisms. Reliance on self-report measures may also have introduced bias, highlighting the value of incorporating objective indicators. Similarly, stronger connections between psychosocial changes and clinical outcomes, such as HbA1c, should be more explored. Finally, participant attrition was not analyzed in depth, and although exclusion criteria were carefully applied in this study and included different conditions, it is possible that other additional health problems/comorbidities not explicitly considered may also influence participants’ psychological well-being. Future research should report dropout patterns and their potential influence on outcomes, as well as take into account a broader range of comorbid conditions to more comprehensively capture the complexity of diabetes and its interaction with mental health outcomes.

Nonetheless, despite these limitations, this study provides encouraging evidence for the feasibility and effectiveness of the 10VIDA program, supporting the need for more rigorous longitudinal research in adolescents with T1DM.

## 5. Conclusions

The present study provides significant insight into the socioemotional status of adolescents living with Type 1 diabetes mellitus (T1DM). Adolescence is a critical developmental stage that poses distinctive challenges in the management of chronic diseases, particularly with regard to psychological well-being and emotional adjustment. In light of these challenges, the 10VIDA program was developed to address these issues by focusing on psychological adaptation, emotional resilience, treatment adherence, and improved quality of life in adolescents with T1DM.

Preliminary findings from the implementation of the 10VIDA program have yielded positive outcomes in various psychosocial domains. The program demonstrated benefits related to reduced perceived disease threat, lower levels of emotional distress, and improvements in overall psychological well-being. The psychological profiles of the study participants revealed varying degrees of anxiety, depression, self-esteem, and illness perception, with marked gender differences. Specifically, girls reported higher levels of anxiety and depression, whereas boys demonstrated better self-esteem and emotional regulation. These findings underscore the complex and nuanced relationship between gender and psychological adjustment in adolescents with T1DM.

The outcomes observed underscore the necessity of integrative psychological care in the treatment of chronic conditions such as T1DM. The 10VIDA program is a noteworthy intervention with the potential to address both the emotional and clinical aspects of chronic disease management. By promoting enhancements in mental health, emotional coping, and disease perception, such interventions have the potential to substantially enhance the quality of life for adolescents grappling with the dual challenges of adolescence and a lifelong medical condition.

The results of this study carry significant implications for clinical practice. They underscore the critical necessity for customized psychosocial support to be integrated within standard diabetes care, particularly during adolescence when emotional vulnerability is exacerbated. The findings further suggest the necessity for gender-sensitive strategies to effectively address the unique emotional and psychological needs of both boys and girls. Multidisciplinary approaches involving psychologists, medical professionals, and families are essential in promoting treatment adherence and overall well-being.

While the 10VIDA program demonstrates considerable potential in supporting adolescents with T1DM, further research is necessary to substantiate these findings. It is imperative that sustained scientific inquiry, extended longitudinal observation, and widespread integration of customized, comprehensive strategies are prioritized to enhance the physical and mental well-being of adolescents grappling with chronic illness such as DMT1.

## 6. Patents

This intervention protocol is registered as intellectual property at the Universitat de València, under the registration number of UV-MET-202131R.

## Figures and Tables

**Table 1:** 10VIDA program summary: sessions and objectives.

10VIDA PROGRAM (Sessions with Patients)		
Session	Theme	Aims
S1. My beliefs	Adjustment to illness	Assess, recognize, and value beliefs, concerns, or fears related to the disease.
S2. A look inside me	Self-esteem/self-concept	To develop behavioral patterns that facilitate an adequate self-image and identity, without the stigmas of illness.
S3. From serenity	Coping with fear	To learn to identify, attend to, and manage the anxious symptomatology associated with the life situations that a chronic disease in adolescence may entail. To favor a serene and positive attitude, knowing their own fears.
S4. Emotions: My friends	Emotional self-regulation	Encourage a coping and resilient attitude to facilitate the acquisition of appropriate habits and behaviors. To promote positive emotions that can cushion the daily situations with the disease.
S5. A look outside	Social area	To reflect on the importance of friendships at this age, and that they are sources of support in the face of illness and treatment.

**Table 2:** Comparison between control and experimental groups post-intervention (Mann–Whitney U test).

Questionnaire	Variable	Group		*Z*	*p*	r
		Control Ar (SD) (N = 9)	Experimental Ar (SD) (N = 10)			
BIEPS-J	Control of situations	8.22 (1.78)	11.60 (1.13)	−1.342	0.75	−0.308 *
	Links	8.06 (2)	11.75 (0.31)	−1.849	0.064 ^	−0.424 *
	Projects	9.50 (2)	10.45 (1.47)	−0.392	0.695	−0.090
	Acceptance	7.67 (2.10)	12.10 (0.84)	−1.821	0.069 ^	−0.418 *
HADS	Depression	11.67 (2.20)	8.50 (1.64)	−1.255	0.209	−0.288
SDQ	SDQSE	10.67 (3.35)	9.40 (2.33)	−0.506	0.613	−0.116
	SDQH	9.89 (2.45)	10.10 (2.87)	−0.084	0.933	−0.019
	SDPRI	10.50 (1.41)	9.55 (2.16)	−0.380	0.704	−0.087
	SDQCP	10.78 (1.22)	9.30 (1.64)	−0.615	0.538	−0.141

Note: Ar = average ranges; SD = standard deviation; *Z* = value of Mann–Whitney U test statistic; *p* = level of significance, * *p* ≤ 0.05 (note: statistical trend = ^ *p* ≤ 0.1); r = effect size (in r 0.1–0.3 = weak effect, 0.3–0.5 = medium effect, >0.5 ** = large effect).

**Table 3:** Comparison between control and experimental groups post-intervention (Student’s *t*-test).

Questionnaire	Variable	Group		*t*	*p*	D
		Control M (SD) (N = 9)	Experimental M (SD) (N = 10)			
CSR	Self-esteem	31.11 (8.17)	32.30 (5.18)	−0.374	0.707	−0.075
BIEPS-J	Total wellness	31.55 (6.65)	35.90(2.46)	−1.972	0.071 ^	−0.511 **
HADS	Anxiety	5.44 (4.50)	3.90(3.38)	0.851	0.407	−0.143
	Overall score	8.33 (6.57)	5.50 (4.79)	1.082	0.294	−0.188
SDQ	SDQPC	1.77(1.20)	2.80 (2.25)	−1.213	0.319	−0.229
	Total score	10.22 (6.28)	10.00 (7.95)	0.067	0.947	−0.038
EP	EP Affection and Communication	42.55 (5.47)	41.70 (6.73)	0.302	0.767	−0.028
	EP Autonomy Promotion	39.11(5.44)	40.50 (4.97)	−0.581	0.564	−0.122
	EP Behavioral Control	29.77 (5.60)	30.10 (5.34)	−0.128	0.899	−0.019
	EP Psychological Control	24.66 (9.74)	25.70 (10.47)	−0.222	0.827	−0.075
	EP Disclosure	25.44 (5.38)	24.40 (4.78)	0.448	0.660	−0.143
	EP Humor	32.22 (3.70)	29.60 (3.50)	1.587	0.131	−0.361 *
BIPQ	Perception of illness	34.55 (13.92)	32.30 (12.03)	0.379	0.709	−0.113
ESCQ	ESCQ Perception and Understanding	4.65 (0.83)	5 (0.55)	−1.086	0.293	−0.170
	ESCQ Expression and Labeling	4.82 (0.89)	4.98 (0.96)	−0.373	0.714	−0.085
	ESCQ Management and Regulation	4.82 (0.82)	4.88 (0.67)	−0.175	0.863	−0.028

Note: M = mean; SD = standard deviation; *t* = value of *t*-test statistic; *p* = level of significance, * *p* ≤ 0.05 and ** *p* ≤ 0.01 (note: statistical trend = ^ *p* ≤ 0.1); d = Cohen’s effect size (in Cohen’s d = small TE ≈ 0.20; moderate TE ≈ 0.50; large TE ≈ 0.80 **).

**Table 4:** Intra-subject study comparison.

Questionnaire	Variable	Group			*Z*	*p*	T1-T2 (r)	T2-T3 (r)	T1-T3 (r)
		T1 //Ar//M (SD)	T2 //Ar//M (SD)	T3 //Ar//M (SD)					
CSR	Self-esteem	//1.96//31.69 (4.83)	//2.04//31.84 (6.05)	//2//32.15 (5.42)	0.043	0.978	0.845 (−0.054)	0.922 (0.027)	0.922 (−0.027)
BIEPS-J	Control of situations	//1.92//10.61 (1.60)	//1.85//10.23 (1.69)	//2.23//10.92 (1.03)	1.600	0.449	0.845 (−0.218)	0.327 (−0.272)	0.433 (0.308 *)
	Links	//2.08//8.61 (0.86)	//1.77//8.07 (1.70)	//2.15//8.69 (0.63)	2.947	0.229	0.433 (0.218)	0.327 (−0.272)	0.845 (−0.054)
	Projects	//2.08//7.38 (1.85)	//1.81//7.15 (1.99)	//2.12//7.76 (1.73)	1.187	0.552	0.492 (0.190)	0.433 (−0.218)	0.922 (−0.027)
	Acceptance	//2.23//8.38 (0.86)	//1.58//7.46 (1.71)	//2.19//8.30 (0.75)	6.276	0.43	0.096 ^ (0.462 *)	0.117 (−0.435 *)	0.922 (0.027)
	General Well-being	//2.15//35 (4.10)	//1.58//32.92 (6.30)	//2.27//35.69 (2.81)	4.537	0.103	0.141 (0.408 *)	0.078 ^ (−0.490 *)	0.769 (−0.082)
HADS	Anxiety	//2.08//4.07 (3.17)	//2.27//5.15 (3.86)	//1.65//4.23 (3.56)	3.045	0.218	0.624 (−0.136)	0.117 (0.435 *)	0.281 (0.299)
	Depression	//2//2.23 (2.80)	//2.27//2.61 (2.06)	//1.73//1.84 (1.81)	3.161	0.206	0.492 (−0.190)	0.170 (0.38 *)	0.492 (0.190)
	Overall score	//2.12//6.30 (5.58)	//2.35//7.76 (5.77)	//1.54//6.07 (5.20)	4.979	0.83	0.556 (−0.163)	0.039 * (0.571 **)	0.141 (0.408 *)
SDQ	SDSE	//2.12//2.69 (2.46)	//2.23//3.53 (3.09)	//1.65//2.30 (2.46)	3.231	0.199	0.769 (−0.082)	0.144 (0.408 *)	0.239 (0.326 *)
	SDQPC	//1.92//1.76 (1.87)	//2.04//2.23 (2.24)	//2.04//2.23 (2.08)	0.250	0.882	0.769 (−0.082)	11 (0.00)	0.769 (−0.082)
	SDQH	//2.19//3.84 (2.40)	//1.92//3.38 (2.84)	//1.88//3.38 (2.87)	1.027	0.598	0.492 (0.190)	0.922 (0.027)	0.433 (0.218)
	SDPRI	//2//1.84 (2.19)	//2.08//2.30 (2.35)	//1.92//1.61 (1.98)	0.267	0.875	0.845 (−0.054)	0.695 (0.109)	0.845 (0.054)
	SDQCP	//2.12//8.61 (1.50)	//1.85//8.53 (1.56)	//2.04//8.46 (1.61)	0.703	0.704	0.492 (0.190)	0.624 (−0.136)	0.845 (0.054)
	Total score	//2.04//10.15 (7.20)	//2.19//11.46 (9.16)	//1.77//9.53 (7.75)	1.37	0.502	0.695 (−0.109)	0.281 (0.299)	0.492 (0.190)
EP	EP Affection and Communication	//2.04//42.69 (4.13)	//2.15//43.15 (4.94)	//1.81//41.61 (5.96)	0.913	0.633	0.769 (−0.082)	0.377 (0.245)	0.556 (0.163)
	EP Autonomy Promotion	//2//40.15 (5.61)	//1.88//40.53 (4.85)	//2.12//40.69 (4.87)	0.360	0.835	0.769 (0.082)	0.556 (−0.163)	0.769 (−0.082)
	EP Behavioral Control	//2.08//30.23 (4.93)	//2//30.30 (4.13)	//1.92//30.38 (4.92)	0.167	0.920	0.845 (0.054)	0.845 (0.054)	0.695 (0.109)
	EP Psychological Control	//2//21.84 (8.08)	//1.96//22.76 (10.63)	//2.04//24.30 (11.33)	−0.039	0.981	0.922 (0.027)	0.845 (−0.054)	0.922 (−0.027)
	EP Disclosure	//1.85//23.76 (4.03)	//2.42//25.92 (3.47)	//1.73//24.53 (4.38)	3.796	0.150	0.141 (−0.408 *)	0.078 ^ (0.490 *)	0.769 (0.082)
	EP Humor	//1.62//30.07 (3.27)	//2.23//31.23 (3.58)	//2.15//29.92 (5.13)	3.304	0.192	0.117 (−0.435 *)	0.845 (0.054)	0.170 (−0.381 *)
BIPQ	Perception of illness	//2.04//36 (9.25)	//1.92//35.53 (11.31)	//2.04//35.30 (12.63)	0.120	0.942	0.769 (0.082)	0.769 (−0.082)	1 (0.0)
ESCQ	ESCQ Perception and Understanding	//1.88//4.85 (0.53)	//1.77//4.78 (0.57)	//2.35//5.03 (0.50)	2.800	0.247	0.769 (0.082)	0.141 (−0.408 *)	0.239 (−0.326 *)
	ESCQ Expression and Labeling	//1.85//4.59 (1.22)	//2.19//4.97 (1.01)	//1.96//4.78 (0.96)	0.933	0.627	0.377 (−0.245)	0.556 (0.163)	0.769 (−0.082)
	ESCQ Management and Regulation	//1.77//4.48 (1.08)	//1.73//4.73 (0.826)	//2.50//4.84 (0.77)	5.080	0.079 ^	0.922 (0.027)	0.050 * (−0.544 **)	0.062 ^ (−0.517 **)

Note: /Ar/ = average ranges; M = mean; SD = standard deviation; *Z*: Value of statistic; *p* = level of significance, * *p* ≤ 0.05 and ** *p* ≤0.01 (note: statistical trend = ^ *p* ≤ 0.1); r = effect size (0.1–0.3 = weak effect, 0.3–0.5 * = medium effect, >0.5 ** = large effect).

## Data Availability

The data presented in this study are available on request from the corresponding author. They are not publicly available due to data privacy and confidentiality.

## Supplementary Materials

The following supporting information can be downloaded at: https://www.mdpi.com/article/10.3390/children12101291/s1, Table S1: descriptive clinical and sociodemographic data of adolescents; Table S2: descriptive analysis; Table S3: main correlations; Table S4: mean comparison by gender (Student’s *t* test); Table S5: main comparison by gender (Mann-Whitney U-test); Table S6: comparison according to presence of comorbidities (Student’s *t* test); Table S7: comparison according to presence of comorbidities (Mann-Whitney U-test).

## Abbreviation

The following abbreviation is used in this manuscript:

T1DM	Type 1 Diabetes Mellitus
