# Supplementary material for: The Impact of the 10VIDA Program on Socioemotional Adjustment and Psychological Well-Being in Adolescents with Type 1 Diabetes Mellitus: A Preliminary Study
Source: Children (Basel). 2025 Sep 24;12(10):1291. doi: 10.3390/children12101291 (PMC12564141; doi:10.3390/children12101291)
Supplement: Supplementary file 1 [file children-12-01291-s001.zip › children-3794718-supplementary.pdf]

SUPPLEMENTARY MATERIAL

Table S1. Descriptive clinical and sociodemographic data of adolescents

		n	%
Gender	Male	11	57.9
	Female	8	42.1
Secondary Disease/Disorder	None	12	63.2
	Organic	6	31.6
	Psychological	1	5.3
Use of adrenaline	Yes	1	5.3
	No	18	94.7
Frequency of specialist visits	Every 3 months	14	73.7
	Every 4 months	2	10.5
	Every 6 months	2	10.5
	Annual	1	5.3

Table S2. Descriptive analysis

	Mean	Median	SD	Minimum	Maximum
Age	13.31	13	1.66	11	17
Time in treatment in months	43.21	33	35.79	5	138
Hospital Admissions	1.68	2.00	1.455	0	6
Duration of hospital admissions	1.05	0	1.580	0	5
Emergency room visits	.84	0	1.834	0	7
Hospitalization derived from emergencies	.21	0	.713	0	3
Self-esteem (CSR)	30.52	31	6.012	20	38
Situation control (BIEPS-J)	10.78	11	1.08	9	12
Psychosocial Links (BIEPS-J)	8.526	9	.904	6	9
Projects (BIEPS-J)	7.473	8	1.67	5	9
Self-acceptance (BIEPS-J)	8.05	8	1.22	4	9
Psychological well-being (BIEPS-J)	34.84	35	3.37	29	39
Clinical Anxiety (HADS)	4.26	4	3.46	0	12
Clinical Depression (HADS)	2.26	1	2.40	0	7
General Emotional Discomfort (HADS)	6.52	6	5.49	0	18
Emotional symptomatology (SDQ)	3	3	2.88	0	8
Behavioral problems (SDQ)	2.15	2	1.89	0	7
Hyperactivity (SDQ)	3.26	3	2.18	0	8
Problems with peer relationships (SDQ)	2.26	2	2.25	0	8
Prosocial Behavior (SDQ)	8.36	9	1.46	5	10
Psychopathology and general emotional adjustment (SDQ)	10.68	10	7.83	1	27
Parental Style: Affection and Communication (EP)	41.36	45	8.96	12	48
Parental Style: Autonomy Promotion (EP)	40.68	42	5.70	26	48
Parental Style: Behavioral Control (EP)	29.89	32	5.81	15	36
Parental Style: Psychological Control (EP)	22.63	24	8.15	8	37
Parental Style: Disclosure (EP)	22.63	24	6.42	5	30
Parental Style: Humor (EP)	30.31	30	5.02	14	36
Perception of illness (BIPQ)	32.79	32	11.04	17	60
Emotional Perception and Comprehension (ESCQ)	4.74	4.85	.672	3.29	5.71
Expression of Emotion and Labels (ESCQ)	4.66	5.14	1.27	1.86	6
Emotion Management and Regulation (ESCQ)	4.57	4.85	.90	2.71	5.43

* SD = Standard deviation.

Table S3. Main correlations

	1	2	3	4	5	6	7	8	9	10	11	12	13	14	15	16	17	18	19	20	21	22
1. Age	1.000																					
2. Time in treatment	0.076	1.000																				
3. Self-esteem (RSE)	-0.234	-0.204	1.000																			
4. Control of situations (BIEPS)	0.035	0.490*	0.163	1.000																		
5. Links (BIEPS)	0.082	0.221	-0.055	0.222	1.000																	
6.Projects (BIEPS)	0.404	0.459*	0.151	0.453	0.426	1.000																
7.Acceptance (BIEPS)	-0.230	0.081	0.558*	0.520*	0.315	0.251	1.000															
8.Total Adolescent Well-Being (BIEPS)	0.161	0.406	0.355	0.692**	0.656**	0.786**	0.673**	1.000														
9 Clinical Anxiety (HADS)	0.131	0.015	-.577**	-0.464*	-0.140	-0.195	-0.714**	-0.501*	1.000													
10 Clinical Depression (HADS)	0.221	-0.115	-0.389	-0.543*	-0.356	-0.274	-0.772**	-0.657**	0.761**	1.000												
11. Emotional Distress (HADS)	0.206	-0.055	-.507*	-0.476*	-0.240	-0.242	-0.729**	-0.568*	0.939**	0.908**	1.000											
12. Emotional Management and Regulation (ESCQ)	-0.235	0.503*	0.163	0.541*	0.404	0.373	0.591**	0.554*	-0.446	-0.618**	-0.587**	1.000										
13. Emotional Perception and Understanding (ESCQ)	0.064	0.135	-0.396	0.036	0.454	0.183	-0.038	0.153	-0.091	-0.165	-0.132	0.110	1.000									
14. Expression and labeling of emotions (ESCQ)	-0.050	0.460*	0.377	0.618**	0.483*	0.513*	0.804**	0.792**	-0.702**	-0.771**	-0.750**	0.751**	0.171	1.000								
15. Perception of illness (BIPQ)	0.478*	-0.308	-0.291	-0.482*	-0.039	-0.034	-0.430	-0.263	0.472*	0.523*	.497*	-0.470*	0.035	-0.480*	1.000							
16. Parental Style: Promoting Autonomy (EP)	-0.164	0.300	0.445	0.668**	0.330	0.491*	0.608**	0.740**	-0.760**	-0.860**	-0.860**	0.620**	0.203	0.719**	-0.486*	1.000						
17. Parental Style: Behavioral Control (EP)	0.350	-0.302	0.443	-0.018	0.049	0.303	0.098	0.203	-0.196	-0.206	-0.235	0.120	-0.264	0.090	0.115	0.244	1.000					
18. Parental Style: Affection and Communication (EP)	0.372	0.324	0.481*	0.445	0.056	.598**	0.384	.568*	-0.317	-0.163	-0.222	0.262	-0.168	0.409	0.024	0.421	0.448	1.000				
19. Parental Style: Disclosure (EP)	0.419	0.083	0.189	-0.088	0.229	.492*	-0.031	0.303	0.071	-0.038	-0.023	0.130	-0.079	0.194	0.395	0.147	.665**	0.371	1.000			
20. Parental Style: Psychological Control (PD)	-0.473*	-0.348	-0.164	-0.362	-0.135	-.562*	-0.129	-.474*	0.080	0.300	0.184	-0.319	-0.224	-0.353	-0.093	-0.433	-0.387	-.533*	-.535*	1.000		
21. Parental Style: Humor (EP)	0.157	0.091	0.176	0.222	-0.107	0.312	0.105	0.284	-0.195	-0.209	-0.228	0.184	-0.087	0.049	0.085	.478*	0.390	.617**	0.282	-0.421	1.000	
22. Psychopatology (SDQ)	0.133	-0.123	-0.549*	-0.433	-0.295	-0.375	-0.563*	-0.569*	0.758**	0.630**	0.725**	-0.372	-0.242	-0.600**	.530*	-0.706**	-0.046	-0.181	0.036	0.217	-0.006	1.000

p = level of significance *p ≤0.05 and **p ≤0.01 (note: statistical trend = ^p ≤0.1

Table S4. Mean comparison by gender (Student´s T test)

Questionnaire	Variable	Genre		t	p	D
		Male<br>M (SD) (n=11)	Female<br>M (SD) (n=8)			
HADS	Anxiety	2.11 (1.83)	6.87 (3.35)	-3.446	0.003**	-1.61**
	Depression	1.11 (2.26)	3.75 (2.26)	-3.287	0.020*	-1.52**
	Overall Score	3.22 (3.45)	10.62 (5.23)	-3.575	0.002**	-1.61**
SDQ	SDQH	2.44 (2.06)	4 (2.13)	-1.425	0.227	0.662*
EP	Autonomy Promotion	43.88 (3.55)	37.50 (6.59)	1.698	0.108	0.789**
	Psychological Control	21.22 (9.35)	23 (7.80)	0.31	0.975	0.015
BIPQ	Perception of illness	26.56 (9.02)	39.75 (10.71)	-2.082	0.053^	-.968**
ESCQ	Perception and Comprehension	4.61 (0.77)	4.85 (.65)	0.159	0.875	0.074

* Note. M = mean; SD = standard deviation; t: value of t-test statistic; p = level of significance *p ≤0.05 and **p ≤0.01 (note: statistical trend = ^p ≤0.1); d= Cohen´s effect size (in Cohen's d = small TE ≈ 0.20; moderate TE ≈ 0.50*; large TE≈ 0.80**).

Table S5. Mean comparison by gender (Mann Whitney U-test)

Questionnaire	Variable	Genre		*Z*	p	*r*
		Male<br>*Ar (SD) (n=11)*	Female<br>*Ar (SD) (n=8)*			
CSR	Self-esteem	12.68 (4.80)	6.31 (5.09)	3.132	0.012*	0.719**
BIEPS-J	Control of situations	12.55 (1)	6.50 (.83)	-2.402	0.020*	-0.551**
	Links	10.55 (1.03)	9.25 (.75)	-0.640	0.522	-0.147
	Projects	10.68 (1.74)	9.06 (1.66)	-0.652	0.515	-0.150
	Acceptance	13.45 (.44)	5.25 (1.35)	-3.357	<0.001**	-0.770**
	General Well Being	12.23 (3.03)	6.94 (2.77)	2.249	0.041*	0.516**
SDQ	Socio Emotional	7.50 (1.78)	13.44 (2.47)	-2.335	0.020*	-0.536**
	SDQPC	8.64 (1.33)	11.88 (1.99)	-1.266	0.206	-0.290
	SDPRI	8.82 (1.41)	11.63 (2.12)	-1.093	0.274	-0.251
	SDQCP	9.55 (1.33)	10.63 (1.60)	-0.428	0.669	-0.098
	Overall Score	8 (4.92)	12.75 (6.94)	-1.792	0.045*	-0.411*
EP	Affection and Communication	11.91 (11.65)	7.38 (6.74)	-1.754	0.079^	-0.402*
	Behavioral Control	10.55 (6.70)	9.25 (5.39)	-0.498	0.618	-0.114
	Disclosure	8.82 (7.46)	11.63(5.93)	-1.077	0.281	-0.247
	Humor	10.14 (6.43)	9.81 (4.05)	-0.404	0.691	-0.093
ESCQ	Expression and Labeling	13.09 (1.30)	5.75 (1.04)	-2.815	0.004**	-0.646**
	EManagement and Regulation	12.77 (0.87)	6.19 (0.76)	2.739	0.009**	0.628**

* Note. Ar=average ranges; SD = standard deviation; Z: value of Mann Whitney U-test statistic p = level of significance *p ≤0.05 and **p ≤0.01 (note: statistical trend = ^p ≤0.1); r=effect size (in r 0.1-0.3=weak effect. 0.3-0.5*=medium effect. >0.5**=large effect)

Table S6. Comparison according to presence of comorbidities (Student´s T test)

Questionnaire	Variable	Presence of additional disease		*t*	*p*	D
		No other disease *M (SD)*	Additional disease *M (SD)*			
RSE	Self-esteem	29.33	32.57 (3.95)	1.31	0.207	0.543**
HADS	Anxiety	4 (2.38)	4.41 (4.05)	-0.246	0.808	-0.112
	Overall Score	6.83 (6.39)	6 (3.87)	-0.311	0.760	-0.148
ESCQ	ESCQ Perception and Understanding	4.65 (0.79)	4.89 (0.37)	0.751	0.463	0.357*
	ESCQ Expression and Labeling	4.59 (1.21)	4.77 (1.47)	0.289	0.776	0.138
EP	EP Affection and Communication	38.83 (10.39)	45.71 (2.87)	1.696	0.108	0.807**
	EP Autonomy Promotion	39.66(6.31)	42.42 (4.35)	1.019	0.323	0.485*
	EP Behavioral Control	28.75(6.53)	31.85 (3.97)	1.133	0.273	0.539**
	EP Psychological Control	25.25(7.89)	18.14 (6.91)	-1.975	0.065^	-0.939**
	EP Disclosure	22.08(7.53)	23.57 (4.23)	0.477	0.640	0.227
BIPQ	Perception of illness	32.75 (12.72)	32.85 (8.31)	0.20	0.984	0.009

* Note. M = mean; SD = standard deviation; t: value of t-test statistic; p = level of significance *p ≤0.05 and **p ≤0.01 (note: statistical trend = ^p ≤0.1); d= Cohen´s effect size (in Cohen's d = small TE ≈ 0.20; moderate TE ≈ 0.50*; large TE≈ 0.80**).

Table S7. Comparison according to presence of comorbidities (Mann Whitney U-test)

Questionnaire	Variable	Presence of disease		Z	p	r
		No other disease Ar (SD)	Additional disease Ar (SD)			
BIEPS	Control of situations	12 (0.99)	8.83 (1.21)	-1.229	0.261	-0.282
	Links	10.86 (0.79)	9.50 (1.13)	-0.655	0.650	-0.150
	Projects	12.29 (1.54)	8.67 (1.95)	-1.423	0.196	-0.326*
	Acceptance	10.67 (1.44)	9.71 (0.75)	-0.316	0.773	-0.072
	General Well Being	11.79 (3)	8.96 (3.93)	-1.073	0.299	-.246
HADS	Depression	1.5 (2.46)	1 (2.44)	-1.229	0.261	-0.341*
ESCQ	Management and Regulation	4.57 (0.86)	5.14 (0.95)	-1.151	0.261	-0.319*
EP	EP Humor	11.29 (5.99)	9.25 (2.49)	-0.765	0.482	-0.176

* Note. Ar=average ranges; SD = standard deviation; Z: value of Mann Whitney U-test statistic p = level of significance *p ≤0.05 and **p ≤0.01 (note: statistical trend = ^p ≤0.1); r=effect size (in r 0.1-0.3=weak effect. 0.3-0.5*=medium effect. >0.5**=large effect)
